# Comparative Evaluation of High-Throughput In Vitro Digestion Methods for Predicting In Vivo Digestibility and Fecal Odor Emissions in Pigs

**DOI:** 10.3390/ani16060918

**Published:** 2026-03-14

**Authors:** Ching-Yi Chen, Ruei-Yang Huang, Han-Tsung Wang

**Affiliations:** Department of Animal Science and Technology, National Taiwan University, Taipei 10672, Taiwan; ronichen@ntu.edu.tw (C.-Y.C.); houng87129@gmail.com (R.-Y.H.)

**Keywords:** in vitro digestion method, dialysis, fermentation kinetics, odorous compounds, digestibility prediction

## Abstract

Accurate and physiologically relevant in vitro models are needed to predict nutrient digestibility and hindgut fermentation in pigs, as conventional in vivo trials are resource-intensive and raise animal welfare concerns. This study evaluated and compared the predictive performance of three in vitro digestion approaches—shaking (S), dialysis (D), and a combined shaking plus dialysis (SD) method—for estimating in vivo apparent total tract digestibility (ATTD) and fermentation characteristics across weaning, growing, and finishing pigs. Commercial diets were subjected to simulated gastric and small-intestinal digestion using S, D, or SD, followed by fecal inoculation to model hindgut fermentation for 12 and 48 h. During the gastrointestinal phase, crude protein digestibility was highest with D (>75%), intermediate with SD, and lowest with S (50–60%), indicating that product removal by dialysis mitigated enzyme inhibition from metabolite accumulation. After 48 h of fermentation, all methods showed strong linear correlations with in vivo ATTD (r > 0.93), but only D achieved high absolute agreement (Lin’s CCC > 0.95 for dry matter and crude protein). Moreover, D and SD at 48 h closely reflected in vivo fecal profiles of skatole, indole, and microbial enzyme activities, with D at 12 h showing an especially strong correlation for protease (r = 0.98). While D provided the most precise predictions of absolute values, the SD method offered an optimal balance between physiological relevance and operational efficiency, supporting its use as a robust, high-throughput platform for porcine feed evaluation and fecal nitrogenous odorant prediction.

## Simple Summary

Predicting how pigs digest feed and produce manure odors usually requires animal trials, which are costly, time-consuming, and raise animal welfare concerns. This study tested three laboratory-based (in vitro) digestion methods—shaking (S), dialysis (D), and a combined shaking plus dialysis (SD)—to see how well they could mimic what actually happens in pigs at different growth stages. After simulating stomach and small-intestinal digestion, we let pig fecal microbes ferment the remaining feed. We found that methods that removed digested products by dialysis (D and SD) better represented real digestion because they prevented build-up of metabolites that can block enzymes. The D method gave the most accurate predictions of whole-tract digestibility, while the SD method offered a practical balance between accuracy and efficiency. Both D and SD closely matched real pig manure in terms of odor-producing compounds and microbial enzyme activities. Overall, the SD method provides a reliable, high-throughput laboratory tool to evaluate pig feeds and predict manure odors, reducing the need for animal testing and helping design diets that improve nutrient use and lessen environmental impacts.

## 1. Introduction

Currently, proximate analysis is the primary method for evaluating dietary nutrients; however, a significant gap exists between chemical composition and the actual efficiency of digestion and absorption. Therefore, an objective assessment of the nutritional value of feed is essential in enhancing feed efficiency. While in vivo experiments provide authentic metabolic data and physiological parameters, they are inherently time-consuming, labor-intensive, and cost-prohibitive, especially when comparing a wide array of dietary treatments [1].

Dynamic in vitro digestion systems have been developed to address animal welfare concerns and costs while achieving high correlation with in vivo results. Although dynamic systems like the TNO Gastro-Intestinal Model (TIM) offer high physiological accuracy, their low throughput—typically one sample per run—limits the large-scale evaluation of diverse feed formulations [2]. To overcome this, a rapid and standardized high-throughput screening platform is essential for systematically ranking candidates. Such a system enhances experimental efficiency and minimizes costs by selecting the most promising combinations for subsequent in vivo validation. Nevertheless, the sophisticated equipment required is expensive and limits throughput, making it difficult to perform simultaneous comparisons of multiple source diets [3]. In contrast, static in vitro digestion systems, despite some physiological simplifications compared to the actual gastrointestinal tract, offer advantages in terms of ease of operation and low equipment costs, making them highly effective for large-scale evaluation trials [4]. Previous studies also demonstrated that static models maintain a robust correlation with in vivo data [4,5,6].

However, a lack of standardization persists across different research teams regarding critical parameters, such as enzyme selection, pH environments, temperature, dialysis bag pore sizes, and the choice between shaking flasks or dialysis bags [7]. This variability complicates the comparison of results across different studies. Furthermore, many simulations of the hindgut fermentation stage rely solely on carbohydrate enzymes and buffer solutions, ignoring the complex microbial fermentation characteristics. This omission prevents an effective assessment of emission issues caused by microbial activity [8]. To address this, Williams et al. [6] utilized swine feces as a microbial inoculum to simulate hindgut fermentation, enabling the collection of gas and fermentation end-products to evaluate nutrient degradation and emission profiles.

In static simulations of the gastric and small intestinal phases, the shaking flask and dialysis bag methods are the most prevalent. However, it remains unclear whether these two techniques yield significant differences in final digestibility results. A comprehensive “stomach–small intestine–large intestine” in vitro model that tracks nutrient disappearance and subsequent emission profiles would provide a more intuitive reference for dietary formulation [9]. To investigate the relationship between simulated swine digestion and odor emissions and to establish a reliable protocol for rapid diet comparison, in this study, we employed three methods—a shaking flask, a dialysis bag, and a combined flask–dialysis approach—to simulate gastric and small intestinal digestion. This was followed by in vitro fermentation at varying time intervals to simulate hindgut fermentation. The goal is to compare the impact of these different methodologies on digestibility and emission data across different growth stages, ultimately developing a rapid and accurate in vitro digestion–fermentation pipeline for high-throughput feed evaluation. By combining these into a hybrid flask–dialysis protocol with optimized fermentation, we establish a standardized platform that ensures high procedural efficiency, analytical precision, and robust predictive capacity for porcine nutrition.

## 2. Materials and Methods

### 2.1. Experimental Diets and Animals

To evaluate the effects of different combinations of in vitro digestion methods and fermentation durations on digestibility and fecal nitrogenous odorant emissions in swine diets, commercial diets for weaning (W1, W2), grower (G1, G2), and finisher (F1, F2) pigs were collected from various commercial farms. The results obtained from the in vitro digestion and fermentation models were compared with the in vivo performance data from the respective source farms. The six farms involved in this study are all integrated farrow-to-finish operations with highly similar management protocols. All facilities utilize environmentally controlled housing designed to commercial industry standards.

All pigs used in this study were Landrace × Yorkshire × Duroc (LYD) crossbreds. The diets for each growth stage were formulated based on Nutrition Requirement of Swine (NRC, 7th Ed) recommendations, with crude protein (CP) levels adjusted according to the specific management requirements of each farm. Exogenous amino acids were supplemented to meet the requirements for standardized ileal digestibility (SID) of essential amino acids. All experimental diets underwent proximate analysis, and the nitrogen-free extract (NFE) content was calculated (%NFE = %DM − %Ash − %Crude Protein − %Crude Fat − %Crude Fiber). The nutrient values of experimental diets and in vivo digestibility results are provided in Table 1.

### 2.2. In Vitro Experiment

All diets were ground and passed through a 20-mesh screen before weighing. The digestion process was based on our previous studies [10], with some modifications in fermentation time length. To standardize the digestion process, the activity of digestion enzymes applied in the process was assayed as described by Minekus et al. [3] before the experiment.

#### 2.2.1. Simulated Digestion Fluids

The gastric fluid buffer (G-buffer: 88.5 mM of NaCl, 6.6 mM of KCl, and 10 mM of HCl) and the intestinal fluid buffer (I-Buffer: 98.7 mM of NaCl, 16.4 mM of KCl, 170 mM NaH_2_PO_4_, and 30 mM Na_2_HPO_4_) were formulated to match the in vivo ionic concentration of gastric and intestinal fluid in growing pigs [5]. The pH of the G-buffer was adjusted to 2.5 with 0.2 N of HCl at 39 °C. The I-Buffer was adjusted to 6.8 with 0.2 N of NaOH or HCl at 39 °C.

For the preparation of the pepsin solution, 0.8 g of pepsin (EC 3.4.23.1, Sigma P7000, >250 units/mg; Sigma-Aldrich, St. Louis, MO, USA) was dissolved in 20 mL of G-buffer and thoroughly mixed prior to use. For the preparation of the pancreatin solution, 8 g of pancreatin (8× USP, Sigma P7547; Sigma-Aldrich, USA) was homogenously suspended in 80 mL of I-buffer, followed by centrifugation at 1500× *g* for 5 min at 4 °C (Model 7000; KOUBOTA, Tokyo, Japan). The supernatant was collected and used for subsequent analyses.

#### 2.2.2. In Vitro Digestion

The in vitro digestion process was categorized into three distinct protocols based on the simulation methods for the gastric and small intestinal phases [5,10]:

##### Shaking Method (S)

The simulation of gastric and small intestinal phases was performed using conical flasks within a shaking incubator. Initially, the gastric stage was commenced by mixing 4 g of feed (n = 12) with 15 mL of G-buffer, supplemented with 4 mL of 0.5 N HCl and 1 mL of pepsin solution. This mixture was incubated at 39 °C for a 4 h duration in a water bath oscillating at 100 rpm. To quench the enzymatic reaction, 3 mL of 3 M NaHCO_3_ was introduced, and the pH was adjusted to 6.8 using either 0.2 N HCl or NaOH. Subsequently, for the enteric phase, 13 mL of pre-warmed (39 °C) I-buffer was incorporated into the flask. A 4 mL volume of pancreatin solution was then precisely delivered via syringe into the shaking flasks. This secondary digestion stage proceeded at 39 °C for an additional 16 h at 100 rpm. To prevent pressure buildup from metabolic gases during the extended incubation, each flask was fitted with a unidirectional check valve.

##### Dialysis Method (D)

A dialysis-based approach was employed to execute the two-stage digestion process within semi-permeable membranes. To start the gastric phase, 4 g of feed samples (12 replicates) were combined with 15 mL of G-buffer, 4 mL of 0.5 N HCl, and 1 mL of pepsin solution. The entire mixture was then transferred into rehydrated 15 cm dialysis tubes (Spectra/Pro 3 RC; MWCO: 1000 Da; diameter: 38 mm; Repligen, Anaheim, CA, USA). These sealed bags were submerged in pre-heated (39 °C) G-buffer at a feed-to-buffer ratio of 1:200 (*w*/*v*), with the external medium circulating at a constant velocity of 120 mL/min. After 4 h of dialysis, the gastric reaction was halted by injecting 3 mL of 3 M NaHCO_3_ into the bags. Before transitioning to the intestinal phase, the tubes were rinsed with warm deionized water. The intestinal environment was then established by adding 13 mL of filtered I-buffer and 4 mL of pancreatin solution to each bag. The subsequent 16 h intestinal digestion utilized the same circulating equipment and buffer-to-feed ratio, with G-buffer being replaced by I-buffer to simulate enteric conditions.

##### Shaking-Dialysis Method (SD)

The gastric phase was conducted in shaking flasks, while for the small intestinal phase, we utilized dialysis bags. All digestion and dialysis processes followed the S and D method, but the digestion content was transferred to the dialysis bag after the 4 h gastric phase of digestion.

After the digestion process, the flask or dialysis bag was moved to an ice bath to stop the reaction and transfer the content into a 50 mL centrifuge tube. The residue was collected via centrifugation at 8000× *g* for 20 min (Model 7000; KOUBOTA, Tokyo, Japan). The precipitate was resolved in 5 mL of distilled water then frozen at –80 °C overnight. All collected samples were dried via lyophilization and stored at 4 °C as the fermentation substrate after weighing.

#### 2.2.3. In Vitro Fermentation Process of Colon-Phase Simulation

##### Fecal Inoculum Collection and Preparation

Fresh fecal samples, used as the microbial inoculum for the in vitro fermentation stage, were collected from the respective farms on the morning of the dietary transition to the experimental diets. For the weaning stage, feces were collected on the day of weaning. For the grower and finisher stages, samples were collected on the day that the pigs were transitioned to their respective grower or finisher rations.

Fresh feces (about 100 g/pig) were collected from three pigs in each of the four designated pens (total of 12 pigs per farm) within one hour post-feeding. To ensure anaerobic integrity, samples were caught in sterile bags before touching the ground and kept at 37 °C. The individual pen samples were then pooled and homogenized in a blender under continuous CO_2_ flushing within 15 min. To ensure accurate correspondence with the fecal collection and correlation analysis from the animal trials, the three fecal samples collected per pen were first pooled to form four distinct individual pen-specific inocula (e.g., A, B, C, and D). A ‘mixed inoculation sample’ was subsequently prepared by pooling one-third (by weight) of each individual pen composite. For the 12-replicate fermentation assay, the inoculation scheme was structured to include two replicates each for Pens A, B, C, and D, and four replicates for the Mixed sample (2A + 2B + 2C + 2D + 4 Mixed).

##### In Vitro Fermentation and Gas Production

Fecal inoculum was prepared by homogenizing freshly voided porcine feces with a pre-heated anaerobic buffer at a 1:5 (*w*/*v*) ratio. The composition of the anaerobic medium followed the formulation established by Williams et al. [6]. To model colonic fermentation, 1.0 g of lyophilized residue derived from the preceding GI digestion phases was combined with the fecal inoculum and anaerobic buffer (1:20:20, *w*/*v*/*v*) within 100 mL serum bottles. These vessels were continuously purged with CO_2_ to maintain an anaerobic environment, reaching a final working volume of 40 mL. The fermentation was performed in a water bath (BH-230D, TE-Lab, Taipei, Taiwan) at 39 °C for 12 or 48 h (six replicates per treatment). Fermented samples were collected and centrifuged at 8000× *g* at 4 °C for 15 min to settle the digested residual for the analysis of dry matter (DM) and CP. The supernatant was further centrifuged at 12,000× *g* at 4 °C for 15 min for the analysis of odorous compounds, ammonia, protease, and urease. All samples for the nitrogenous odorants assay were kept at –70 °C before being analyzed, but samples for testing ammonia and enzyme activity (protease and urease) were kept on ice and analyzed immediately. For the nitrogenous odorants assay, one milliliter of the supernatant fraction from the second centrifugation was mixed with 3 mL acetonitrile, and the supernatant was collected after another centrifugation at 13,500× *g* at 4 °C for 10 min.

Real-time monitoring of cumulative gas output was performed exclusively for the 48 h treatment cohort using a wireless ANKOM RF Gas Production System (ANKOM Technology Corp., Fairport, NY, USA), with each bottle equipped with a dedicated pressure sensor module. Upon completion of the 48-h interval, the gas accumulation kinetics were modeled using the multi-phasic equation described by Bauer et al. [11].

At the end of the incubation period (48 h), the cumulative gas production data were fitted to the multiphasic model described by Bauer et al. [11] as follows:

G = A/[1 + (C/t)^B^]

where G is the total gas produced, A is the asymptotic gas production (mL), B is the switching characteristic of the curve, C is the time at which half of the asymptote has been reached (T_1/2_, h), and t is the time (h).

The maximum rate of gas production (R_max_, h^−1^) and the time at which it occurs (T_max_, h) was calculated according to the following equations:

R_max_ = (A × (C)^B^ × [T_max_^(−B−1)^])/(1 + (C^B^) × [T_max_^(−B)^])^2^

T_max_ = C × ([(B − 1)/(B + 1)]^1/B^)

In this model: A value represents the total volume of fermentable substrate. B value reflects the adaptation of the microbial community to the substrate and the rate of fermentation acceleration. The smaller C value signifies a faster overall fermentation process. The R_max_ value indicates high substrate accessibility and concentrated energy release. The T_max_ value reflects substrate complexity, where higher values indicate that microbes require more time to initiate and complete the fermentation process.

### 2.3. In Vivo Feeding Trial and Digestibility Determination

Animal trials were conducted at the source commercial farms. In each trial, pigs within the same building were of the same age and fed identical diets. Each pen containing approximately 30 pigs (totaling ~120 pigs involved in experiment per farm), depending on the specific management practices of the farm. The initial body weights of the pigs at the start of the feeding trials were 6.7 ± 0.4 kg for weaned pigs, 28.3 ± 0.7 kg for grower pigs, and 58 ± 1.3 kg for finisher pigs (Mean ± SE). Feed was provided twice daily (08:00 and 17:00), water was provided ad libitum, and four non-adjacent pens within the same building were selected for sampling to ensure statistical independence. Regarding the statistical unit, the pen was defined as the experimental unit for in vivo analysis (4 pens per farm). For the in vitro-in vivo correlation, we utilized a matched-sample strategy. Diets were sampled from individual feeders across the four pens (e.g., A, B, C, D) and two mixed feed samples from four pens, resulting in 6 distinct diet samples. A total of 36 samples (6 diets with 6 replicates each) were subjected to a multi-stage in vitro digestion procedure.

After a four-week feeding period, three fresh fecal samples (avoiding floor contact) were collected from each pen one hour after the morning feeding. Daily feed intake was recorded for three consecutive days before the morning feeding prior to fecal sampling, and representative feed samples were collected from the site to calculate the average feed intake per pig within each pen. The apparent total tract digestibility (ATTD) of nutrients was determined using acid-insoluble ash (AIA) as an internal marker, following the methodology described by Prawirodigdo et al. [12]. NH_3_-N and enzyme activity (protease and urease) in fecal samples were assayed immediately after collection, and the residual fecal samples were stored at –20 °C for odorous compound assays. Before the odorous assay, about 1 g of the fecal sample was added to the 15 mL test tube and mixed with 9 mL phosphate-buffered saline (pH 7.4). After sonication for 30 min at 4 °C, the sample was centrifuged at 12,000× *g* at 4 °C for 15 min for the analysis of odorous compounds, ammonia, protease, and urease.

All animal care procedures followed in this study were approved by the Institutional Animal Care and Use Committee of the National Taiwan University (approval no: NTU-107-EL-00056).

### 2.4. Chemical Analysis

Proximate analysis of the feed ingredients was conducted following the standardized protocols of the Association of Official Analytical Chemists (AOAC) [13]. Specifically, we determined the content of dry matter (DM, 934.01), crude protein (CP, 954.01), ether extract (EE, 920.39), ash (942.05), crude fiber (CF, 962.09), and acid-insoluble ash (AIA, 941.12). The concentration of ammonia-N was measured via a colorimetric approach based on the phenol–hypochlorite reaction [14]. For the nitrogenous odorants assay (exclude NH_3_), the thawed sample (10 μL) was analyzed using HPLC with a fluorescence detector (FP-4020, JASCO, Easton, MD, USA). Compounds were separated using a LiChrospher 100 RP-18 (5 μm) column (Merck, Burlington, MA, USA). The mobile phase was a mixture of 200 mM ammonium formate and acetonitrile (52:48), the flow rate was 0.5 mL/min, the column temperature was set at 30 °C, and the excitation and emission wavelengths of the detector were 270 and 340 nm, respectively.

Protease activity in the fecal and fermentation samples was assessed by monitoring the liberation of the azo-group from 0.8% azocasein (Sigma 11610; Sigma-Aldrich, USA) after a 30 min incubation at 39 °C [15]. The enzymatic reaction was quenched with 10% trichloroacetic acid on ice, and the absorbance of the resulting supernatant was recorded at 450 nm post-centrifugation. Urease activity was determined using a dedicated assay kit (K378; Biovision, Milpitas, CA, USA) following the manufacturer’s instructions. Furthermore, the total protein concentration in the fermentation and fecal fluids was quantified using Bradford assay reagents (Sigma B6916; Sigma-Aldrich, USA).

### 2.5. Statistical Analysis

Data were analyzed as a 3 × 2 factorial arrangement using the General Linear Model (GLM) procedure of SAS 14.1 (SAS Institute Inc., Cary, NC, USA). T The statistical model was constructed to assess the primary influences of the digestion technique and incubation period, along with their potential interactions. Significance was pre-set at *p* < 0.05, and where necessary, treatment means were differentiated using the Least Significant Difference (LSD) test.

For metabolites, we calculated Pearson’s correlation and analyzed the classification of means based on the difference-of-least-square-means method, following the CORR procedures of the SAS. The REG procedure of SAS was used to develop regression equations for in vitro and in vivo parameters. The probability level (*p* < 0.05) was identified as statistically significant for all variables. To further evaluate the degree of agreement between in vitro digestibility and in vivo results, the Concordance Correlation Coefficient (CCC) was employed [16]. Following the criteria proposed by McBride [17], the consistency was categorized as poor (CCC < 0.90), moderate (CCC = 0.90–0.95), or high (CCC > 0.95).

## 3. Results

### 3.1. Dietary Composition and Apparent Total Tract Digestibility (ATTD)

The chemical compositions of the commercial diets used across the three physiological phases, along with the ATTD observed 4 weeks post-feeding, are summarized in Table 1. Crude protein (CP) content ranged from 18 to 20% for weaning diets, 17 to 18% for grower diets, and 15 to 17% for finisher diets. The dietary formulations were primarily corn–soybean-meal-based, with weaning diets containing 5–6% fish meal and 4–5% whey protein. Based on acid-insoluble ash (AIA) as an internal marker, the ATTD of dry matter (DM) exceeded 80% for all diets. While the CP digestibility in weaning diets reached above 82%, no significant differences were observed between the two dietary treatments within the same physiological phase for either DM or CP digestibility (Table 2).

#### 3.1.1. Effect of Digestion Method on In Vitro Dry Matter Digestibility (IVDMD)

**Figure 1:**
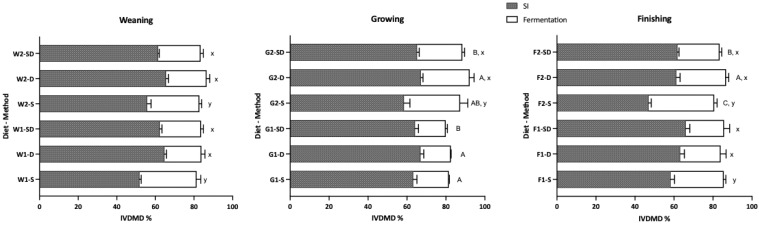
Effects of different digestion methods on in vitro dry matter digestibility (IVDMD) following 48 h of fermentation. Digestion methods: S = Shaking; D = Dialysis; SD = Shaking (gastric phase) + Dialysis (intestinal phase). SI: combined gastric and small intestinal stages. ^A,B,C^ Means within a diet (total tract digestibility) with different superscripts differ significantly (*p* < 0.05). ^x,y^ Means within a diet (fermentation stage digestibility) with different superscripts differ significantly (*p* < 0.05). Data are presented as Mean ± SD.

The IVDMD of diets across three phases, which were simulated via the shaking method (S), dialysis method (D), and combined method (SD) for the gastric and small intestinal stages, followed by fecal inoculation for hindgut fermentation, is shown in Figure 1. While the digestion method did not affect total tract IVDMD in weaning pigs, the S method resulted in significantly higher fermentative digestibility compared to the other two methods. Similarly, for growing and finishing diets, fermentative digestibility was significantly higher in S-treated samples. Across all phases, the S method exhibited lower digestibility during the simulated gastric and small intestinal phases, a trend consistent across diets with different CP levels. Notably, when DM digestibility in the GI phase was significantly lower, the final total tract IVDMD showed minimal variation across methods, primarily due to a compensatory increase in the contribution of the fermentation phase.

#### 3.1.2. Effect of Digestion Method on In Vitro Crude Protein Digestibility (IVCPD)

**Figure 2:**
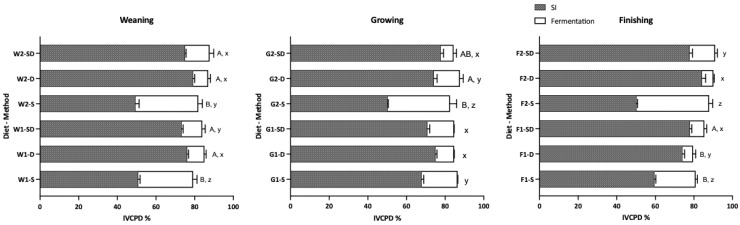
Effects of different digestion methods on in vitro crude protein digestibility (IVCPD) following 48 h of fermentation. Digestion methods: S = Shaking; D = Dialysis; SD = Shaking (gastric phase) + Dialysis (intestinal phase). SI: combined gastric and small intestinal stages. ^A,B^ Means within a diet (total tract digestibility) with different superscripts differ significantly (*p* < 0.05). ^x,y,z^ Means within a diet (fermentation stage digestibility) with different superscripts differ significantly (*p* < 0.05). Data are presented as Mean ± SD.

The IVCPD of residues following fecal fermentation is presented in Figure 2. Similarly to the IVDMD trends, the S method yielded significantly lower CP digestibility during the simulated gastric and small intestinal phases compared to the D and SD methods. For both weaning diets, the D and SD methods resulted in higher total tract IVCPD regardless of dietary CP levels. During the simulated GI phase, CP digestibility for the D and SD methods generally reached above 75%, whereas the S method only achieved 50–60%. This lower enzymatic digestion in the S method led to significantly higher CP degradation during the fermentation process, which subsequently influenced the composition of fermentation metabolites.

### 3.2. Effect of Digestion Method on Concentration of Malodorous Compounds

The production of odorous compounds during fecal inoculation fermentation following the three digestion methods is detailed in Table 3. The digestion method significantly influenced the generation of all nitrogenous malodorous compounds, and fermentation time had a significant effect on odor production in finisher diets. After 48 h of fermentation, residues from the D method exhibited the lowest concentrations of odorants, while the S method yielded the highest; the SD method produced results similar to the D method. Specifically, indole and skatole concentrations were lowest when using the D method. However, regarding p-cresol and ammonia (NH_3_) concentrations, the differences between the D and SD methods were negligible. These results indicate that the higher protein fermentation rate following S digestion resulted in odorant concentrations 1.5 to 2 times higher than the other methods. Furthermore, an inverse relationship was observed between simulated GI CP digestibility and the concentration of nitrogenous odorants. The correlations between gastrointestinal GI CP digestibility and the production of nitrogenous odorants were as follows: p-Cresol (r = −0.5957, *p* = 0.0093), Indole (r = −0.6454, *p* = 0.0047), Skatole (r = −0.6409, *p* = 0.0050), NH_3_ (r = −0.7177, *p* = 0.0013).

### 3.3. Effect of Digestion Method on Gas Production (GP) Kinetics

**Figure 3:**
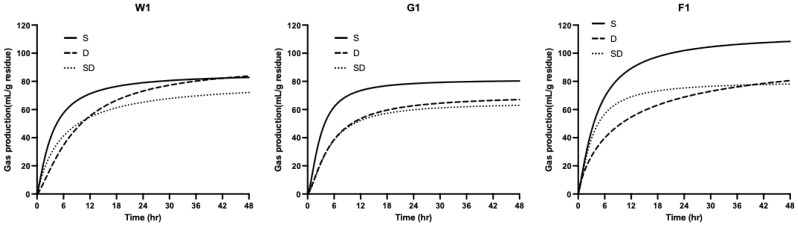

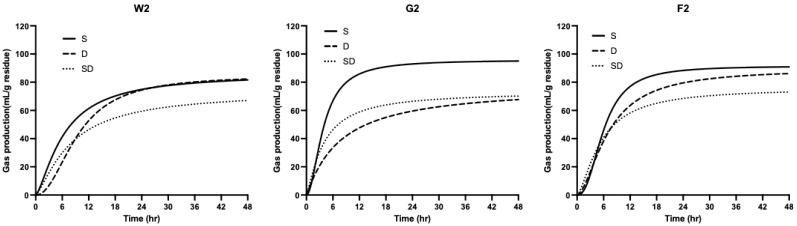
Gas production curves during in vitro fermentation of residues derived from different digestion methods. Digestion methods: S = Shaking; D = Dialysis; SD = Shaking (gastric phase) + Dialysis (intestinal phase). W1, W2 = weaning diets; G1, G2 = growing diets; F1, F2 = finishing diets.

In vitro fermentation GP curves are shown in Figure 3. Residues from the S method resulted in higher total GP and initial GP rates, aligning with the fermentative IVDMD trends in Figure 1. Despite only a 1% difference in CP and similar NFE content between the two grower diets, the S method resulted in a 15% discrepancy in GP. For finisher diets, the F2 diet exhibited higher fermentative IVDMD than the F1 diet under S method digestion, but its GP was lower than F1. Kinetic parameters (Table 4) showed that the S method resulted in the highest asymptotic gas production (A value). In contrast, the D method resulted in the highest time to reach half of the maximum gas production (C value), indicating a significantly slower fermentation rate. No consistent trends were observed for R_max_ and T_max_ across methods. Since a constant substrate weight was used, these results confirm that digestion methods significantly alter residue composition. The removal of products via dialysis significantly impacts fermentation kinetics and metabolite profiles. Correlation analysis revealed that R_max_ and T_max_ were significantly negatively correlated with in vitro GI DM digestibility and highly positively correlated with in vitro fermentative CP digestibility.

### 3.4. Correlation Between In Vivo and In Vitro Digestibility

Correlation analysis between in vivo ATTD of DM and IVDMD across different methods and fermentation times showed that, except for the SD method at 12 h, all treatments exhibited significant positive correlations (r > 0.9) (Table 5). Based on Lin’s Concordance Correlation Coefficient (CCC), the D method at 12 h and 24 h fermentation achieved CCC values above 0.97, demonstrating high consistency with actual in vivo values. The SD method at 48 h also provided a robust estimate (CCC = 0.9530). For CP digestibility (Table 5), 12 h of fermentation yielded a maximum correlation of only 0.85, with poor predictive accuracy (CCC < 0.9). However, after 48 h of fermentation, IVCPD for all three methods correlated highly with in vivo results (r > 0.93). Specifically, the D method at 48 h accurately predicted in vivo CP digestibility (CCC = 0.9520). Overall, the D and SD methods combined with 48 h of fermentation provide highly accurate predictions for in vivo DM and CP digestibility, whereas the S method is less effective in predicting absolute in vivo values.

### 3.5. Correlation of Odorous Compounds and Enzyme Activities In Vivo vs. In Vitro

**Figure 4:**
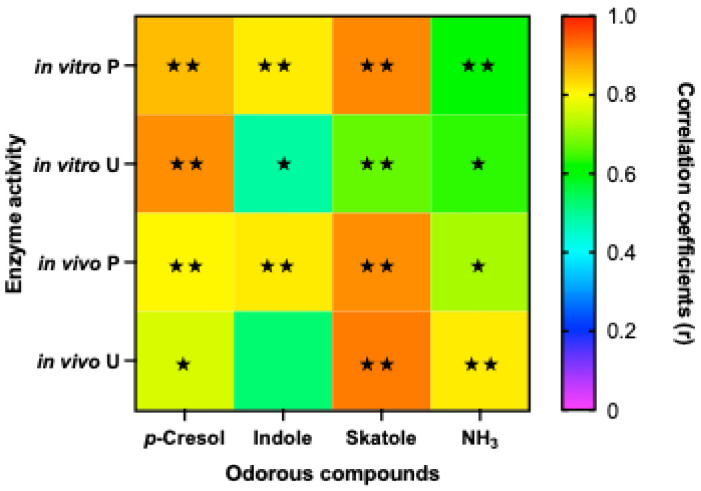
Correlation analysis between odorous compounds and enzyme activity (P: protease; U: urease) in in vitro fermentation samples (48 h) and in vivo fecal samples. Significant correlations are indicated by ★ (*p* < 0.05) and ★★ (*p* < 0.01).

The correlation analysis between odorous compounds in feces from animal trials and those in the fermentation liquid from in vitro trials is illustrated in Figure 4. Following 12 h of fermentation, the concentrations of odorous compounds in the fermentation fluid from the S method showed a strong correlation with those in vivo (r value approx 0.8). In contrast, the D and SD methods required 48 h of fermentation to reach high levels of correlation with fecal nitrogenous odorants. When using the D method with 48 h of fermentation, all odorous compounds except for p-cresol exhibited significant positive correlations with their in vivo counterparts.

Regarding enzymatic activities, the correlation between fecal and in vitro protease and urease activities varied (Figure 4). Protease activity following D digestion and 12 h of fermentation showed the highest in vivo–in vitro correlation (r = 0.98). However, urease activity in the fermentation broth at 12 h showed a weak correlation with fecal urease. Although extending the fermentation time to 48 h did not further improve the correlation for protease activity, it enhanced the correlation for urease activity to r = 0.85 when using the dialysis method. These results suggest that, to achieve an effective assessment across all enzyme activities, the D method paired with 48 h of fermentation provides the most accurate estimation.

### 3.6. Relationship Between Enzyme Activities and Odorous Compound Concentrations

**Figure 5:**
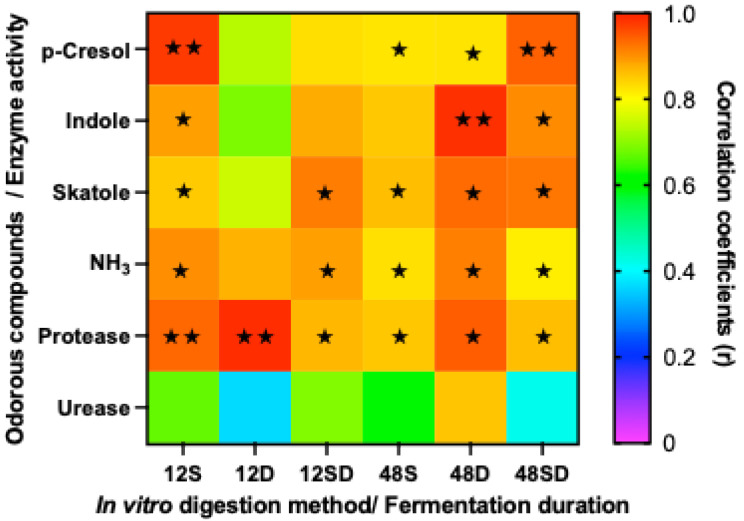
Correlation analysis between odorous compounds and enzyme activity across different digestion methods and fermentation durations. Digestion methods: S = Shaking; D = Dialysis; SD = Shaking (gastric phase) + Dialysis (intestinal phase). Fermentation intervals: 12 h and 48 h. Significant correlations are indicated by ★ (*p* < 0.05) and ★★ (*p* < 0.01).

The relationships between protease and urease activities and specific odorous compounds across both in vivo and in vitro trials are presented in Figure 5. Protease activity in both feces and fermentation broth was significantly and positively correlated with odorant concentrations. Integrating data from both protease and urease activities proved highly valuable in assessing fecal skatole concentrations. For ammonia (NH_3_) concentrations, however, fecal urease activity remained the most reliable predictor.

### 3.7. Impact of Fermentative Digestibility on Odor Production and Enzyme Activity

Further analysis of the effects of residues from different digestion methods on odor production during in vitro fermentation indicated that fermentative crude protein digestibility (IVFCPD) was positively correlated with both odorant concentrations and enzyme activities. Residues processed via the S method exhibited higher variability in fermentative digestibility and odor production. Conversely, the D and SD methods showed highly consistent trends and stronger correlations (Table 6). Notably, fermentative CP digestibility was a superior predictor of fecal nitrogenous odorants compared to fermentative dry matter (DM) digestibility. Therefore, fermentative CP digestibility could be utilized to predict fecal nitrogenous odorants trends, with the D and SD methods providing higher predictive accuracy.

## 4. Discussion

### 4.1. Influence of In Vitro Digestion Methods on Digestibility

In this study, we evaluated three in vitro digestion methods (S, D, and SD) across diets for different growth stages. Despite significant variations in dietary composition, the results for in vitro dry matter digestibility (IVDMD) and in vitro crude protein digestibility (IVCPD) consistently showed that the Dialysis method (D) and Combined method (SD) outperformed the Shaking method (S). Notably, during the simulated gastric and small intestinal phases, S digestion yielded significantly lower DM and CP digestibility than D or SD, with the disparity being more pronounced for CP. This discrepancy is primarily attributed to the removal of digestion metabolites; the dialysis process facilitates the removal of small-molecule products generated through enzymatic hydrolysis, thereby mitigating product inhibition [18]. Since the stomach primarily functions in digestion with negligible nutrient absorption in pigs [19], the digestibility values for D and SD were relatively similar during the simulated upper gastrointestinal (GI) phase.

While the S method resulted in a 10–20% lower CP digestibility in the upper GI simulation compared to the other two methods, the difference in final total tract IVCPD was narrowed to less than 5%. This is explained by the significantly higher fermentative digestibility in S-treated residues (Figure 2), which also exhibited faster gas production rates and higher total gas volume (Figure 3). Previous research has shown that, when adjusting dietary protein levels or sources, shaking digestion methods often result in lower enzymatic digestibility, but the gap narrows after fecal inoculation, as the undigested “bypass” protein becomes a substrate for fermentation [10,19].

Comparing in vivo ATTD with in vitro results, we found that IVDMD correlated highly with in vivo values after only 12 h of fermentation, whereas IVCPD required longer durations. Based on Lin’s Concordance Correlation Coefficient (CCC) analysis, only the Dialysis method (D) provided a consistent and accurate estimate of actual in vivo DM digestibility (CCC > 0.95). In contrast, 12 h fermentation was insufficient for accurately predicting CP digestibility. Our findings align with [20], who noted that the shaking method digestion efficacy varies significantly depending on the dietary fiber matrix. Overall, both D and SD methods paired with 48 h of fermentation show high correlation (r > 0.95) for DM. However, only the D method provides the precision required to simultaneously predict absolute in vivo values for both DM and CP.

### 4.2. Effects of Digestion Methods on Fermentation Dynamics and Metabolites

The fermentation gas production curves revealed that S method-digested residues showed a higher initial gas production rate and required a shorter half-time (C value) to reach maximum gas production. This suggests that S-treated residues retain more rapidly fermentable components, stimulating microbial growth and activity. Although diets W1 and W2 showed similar GI and total tract digestibility, their fermentation kinetics differed. The result indicated that W1 residues fermented faster (shorter T_max_) than W2. Through the simulation of nutrient removal via dialysis, the proportion of insoluble substances in the residue increases, allowing fermentation kinetics to effectively distinguish between dietary residue characteristics [21,22]. However, the inconsistencies in gas parameters between D and SD for the same diet suggest subtle differences in how these methods impact dietary breakdown. While some studies suggest that gas production (A value) can predict ATTD for high-fiber ingredients [20], our results indicate that in high-NFE diets, the digestion method’s influence on residue composition makes gas production an inconsistent predictor of in vivo ATTD. In previous established protocols [20], residues are recovered using filter bags, which may lead to a significant loss of soluble fermented fractions. In contrast, our study incorporates undigested but non-absorbed soluble residues into the fermentation phase, providing a more comprehensive reflection of the actual fermentable load compared to traditional filtration methods.

The digestion method also significantly impacted the production of odorous compounds. Increased fermentation time led to higher odorant accumulation as microbes had more time to metabolize the substrate. However, this accumulation was non-linear, likely due to shifting microbial activity within a closed system. Once fermentable carbohydrates are exhausted, microbes shift toward proteolytic fermentation to obtain energy [23]. Ammonia (NH_3_) is a direct product of microbial protein metabolism. In this study, diets with lower gastric–intestinal CP digestibility showed a more pronounced increase in NH_3_ from 12 to 48 h of fermentation due to the higher residual nitrogen load. This mirrors animal studies where improving protein digestibility or reducing dietary protein via crystalline amino acid supplementation reduces the undigested protein pool in the hindgut, thereby lowering odor emissions [24,25].

We calculated the in vitro fermentative CP digestibility (IVFCPD) and found it to be positively correlated with all odorous compounds. While the S method showed insignificant correlations, the SD and D methods exhibited significant positive correlations (r > 0.9) for most odorants except indole. Furthermore, protease and urease activities in D and SD treatments were highly correlated with IVFCPD (r > 0.85). Interestingly, IVFDMD alone was a poor predictor of most odorants, except for NH_3_ under the D method. This emphasizes that, while total DM and CP digestibility trends may be similar across methods, the specific composition and concentration of residual protein are the primary drivers of microbial odor production [26,27].

Our results support the strategy of lowering dietary CP to reduce nitrogenous emissions. While diets W2 and F2 (with higher CP%) generally produced higher odorant concentrations after digestion compared to the other diet from the same feeding stage (W1 and F1), the S method consistently yielded higher concentrations than D or SD. In the case of diet G2, despite having lower CP%, it produced higher skatole and NH_3_, likely due to its specific protein composition and lower GI digestibility. This confirms that, when the ileal utilization of protein improves, hindgut microbial degradation of urea and protein decreases, effectively slowing ammonia production [14]. In vitro experiments also showed that the GI digestibility of the G2 was lower than G1, and the ATTD of DM and CP in animal experiments was also lower than that in the G1 group.

### 4.3. Correlation Between In Vivo and In Vitro Results

In comparing single ingredients with mixed diets (complete diets), previous studies have highlighted that nutrient interactions during digestion—such as the synergistic or antagonistic effects between different feed components—can significantly alter breakdown patterns [28]. Consequently, the digestibility and fermentation results of individual ingredients cannot be simply extrapolated to predict the performance of a complete diet through additive calculations [29], leading to potential inaccuracies in predicting fermentation metabolites.

In this study, we applied residues obtained from different digestion methods to evaluate fermentation dynamics and product concentrations at various time intervals. The correlation analysis of odorous compound concentrations (Figure 4) and digestibility (Figure 5) indicates that a 48 h fermentation period provides superior correlation with in vivo data compared to a 12 h period. This finding aligns with the work of Graham et al. [30] and Löwgren et al. [31], who demonstrated that 48 h fermentation using either ileal digesta or fecal inocula can yield a robust prediction of digestibility for swine diets with varying fiber content. Similarly, in corn–soybean-meal-based diets supplemented with high-fiber ingredients, in vitro DM digestibility during fecal fermentation has shown high correlation (R^2^ = 0.8–0.9) with in vivo apparent digestibility [20].

The results also demonstrate that the Dialysis (D) method, which simulates nutrient absorption, produces residues that yield higher correlations for all odorous compounds compared to the Shaking (S) method. This consistency with previous studies involving varying dietary CP levels confirms the superiority of dialysis-based models [10]. However, it is noteworthy that prolonged fecal incubation (over 24 h) may lead to product inhibition or a decline in microbial populations within a closed incubation system, potentially causing the in vitro metabolite profile to diverge from actual fecal compositions [32,33].

### 4.4. Enzyme Activities as Predictive Markers for Odor Production

Consistent with other animal trials adjusting dietary protein concentrations, our results show that fecal protease activity is highly correlated with odorant concentrations, particularly skatole, suggesting its potential as a predictive marker [15,34]. In both our in vivo and in vitro assessments, protease activity exhibited a strong positive correlation (r > 0.9) with skatole. Interestingly, while most odorants in the 48 h D-method fermentation correlated well with fecal samples, p-cresol showed a weaker correlation. This may be explained by the specific metabolic pathway of p-cresol, which is derived from tyrosine degradation by specific taxa such as Lactobacillus and Clostridium [35]. The 48 h static fermentation window might not fully capture the slow-turnover kinetics of tyrosine-degrading bacteria compared to the more rapid tryptophan-to-indole pathway, or the dialysis process might have removed certain precursors required for optimal p-cresol synthesis. The weaker correlation for p-cresol in vivo vs. in vitro is also due to its metabolic fate. In the pig’s large intestine, most p-cresol is rapidly absorbed into the bloodstream and transported to the liver, where it is conjugated into p-cresyl sulfate (PCS) or p-cresyl glucuronide (PCG) and excreted via urine. Only a small, non-absorbed fraction remains in the feces, which explains the lower correlation with in vitro production where no absorption occurs [36].

The correlation between odorant and enzyme activity variation likely stems from the dynamic shifts in bacterial populations during in vitro incubation, which fluctuate according to the composition of the dietary residue [37]. Recent studies involving probiotic supplementation have shown that microbial relative abundance shifts significantly at both the phylum and genus levels after 12 and 24 h of cultivation. Although Firmicutes and Bacteroidetes remain the dominant phyla, their specific proportions vary by treatment [38]. Such shifts, particularly toward a more saccharolytic and less proteolytic community, fundamentally alter the patterns of malodorous compound production [39].

### 4.5. Physiological Considerations for Model Optimization

While studies have utilized different dialysis durations, the consensus remains that dialysis-linked simulations offer superior correlation and predictive value for in vivo apparent digestibility [40,41]. In our experiment, we applied a standardized digestion time for all growth phases. However, from a physiological standpoint, the transit time in the digestive tract of weaning pigs is significantly shorter than that of grower or finisher pigs [42]. Therefore, future iterations of this model should consider age-specific adjustments to digestion residence times to further enhance the accuracy of simulations for younger animals.

In this study, 36 samples were processed simultaneously per run (12 replicates per method: S, D, and SD), facilitating robust comparisons while mitigating batch-to-batch enzymatic variations and operational errors. The fermentation phase utilized a single water bath for uniform temperature control and a single, pooled batch of fresh fecal inoculum. This integrated strategy effectively minimized biological variability from donor sources and enhanced the overall precision of the comparative dietary evaluation.

### 4.6. Research Limitations and Contributions to Standardization Beyond Comparing Methods

The standardized in vitro platform developed in this study aims to balance high-throughput screening with physiological relevance, yet several constraints must be considered. Although the low molecular weight cutting dialysis membrane effectively simulates the selective absorption of small molecules (e.g., reducing sugars, free amino acids, and small peptides) while preventing the erroneous loss of larger proteins, the system remains a static simulation.

Another limitation is the absence of microbiota profiling. Without genomic analysis of the fecal microbial composition, the interpretation of fermentation variability is constrained. Results must rely primarily on fermentation end-products and gas production kinetics rather than specific microbial population shifts. While the use of fresh fecal inoculum from healthy donors, pre-monitored for pathogens and total bacterial counts, helped establish a functional baseline, inherent biological variability among different donor sources remains a challenge for absolute standardization.

While the high-throughput capacity to process over 30 samples per run significantly enhances efficiency and mitigates batch-to-batch variations compared to dynamic models like the TNO Gastro-Intestinal Model (TIM), the current system still lacks the high physiological accuracy associated with dynamic kinetic simulations. The reliance on a 48 h fermentation endpoint—justified by stabilized gas production curves—provides high correlation with in vivo data but may not capture the rapid dynamic transitions occurring within the live porcine gut. Although the Concordance Correlation Coefficient (CCC) was calculated using a dataset encompassing six distinct commercial diets, the predictive robustness is inherently tied to the specific microbial and dietary characteristics of the participating farms. While data in this study were limited to six distinct farm sources, the analysis accounted for the risk of overestimating predictive strength by implementing robust statistical measures.

These limitations suggest that while the platform is a reliable tool for ranking feed candidates, future integration with microbial sequencing would provide a more comprehensive mechanistic understanding.

This study establishes a standardized in vitro framework that mimics porcine physiology while ensuring operational stability through the use of consistent buffer systems and commercial enzymes. By employing fresh, farm-specific fecal inoculum, the platform captures a representative microbial profile essential for accurate fermentation assessment. This integrated approach enables the concurrent evaluation of nutrient digestibility and nitrogenous odorant emissions, providing a reliable and efficient tool for the comparative screening of diverse swine diets. This study contributes a rapid, stable, and standardized in vitro digestion and fermentation platform that closely mirrors the physiological conditions of most swine growth stages. The primary advantage of this system is its capacity for multi-objective evaluation; it allows for the concurrent assessment of nutrient digestibility and the emission potential of fecal nitrogenous odorants. This dual-indicator approach provides a more holistic and efficient framework for the comparative evaluation of diverse feed formulations.

## 5. Conclusions

Overall, in vitro dry matter digestibility followed the order D = SD > S, whereas crude protein digestibility ranked D > SD > S, and these differences in the initial enzymatic phase influenced subsequent hindgut fermentation patterns. When considering simulated total tract digestibility, these differences were less marked. After 48 h of fermentation, all three methods showed strong correlations with in vivo apparent digestibility, but only the Dialysis (D) method accurately predicted absolute in vivo values. For fermentative metabolites, the Shaking (S) method correlated well with fecal nitrogenous odorants and protease activity at 12 h; however, for a comprehensive and reliable prediction of both malodorous compounds and microbial enzyme activities, the D and SD methods combined with 48 h of fermentation were superior. Although D provided the highest precision, the SD method integrates the efficient mixing of shaking flasks for gastric digestion with the physiological accuracy of dialysis bags for intestinal absorption. By avoiding the labor-intensive handling of dialysis membranes during the early stages, this hybrid approach markedly increases sample throughput and reduces operational errors. Unlike simple centrifugation, the transition to dialysis after gastric phase allows for the continuous removal of end-products, better mimicking intestinal absorption and preventing the inhibition of enzymatic kinetics.

## Figures and Tables

**Table 1:** Composition assay result of experiment diets.

Composition (%DM)	Weaning		Growing		Finishing	
	W1	W2	G1	G2	F1	F2
Dry matter	87.65	87.92	91.52	90.44	92.96	89.69
Ash	4.70	5.07	5.79	5.56	4.36	5.36
Crude protein	18.17	20.65	18.47	17.67	15.65	17.69
Crude fiber	2.49	2.64	2.76	3.03	3.21	3.16
Crude fat	4.28	4.46	3.50	3.94	3.32	4.39
NFE *	70.36	67.18	69.48	69.80	73.46	69.40

* Nitrogen free extract, %NFE = %DM − (%Ash + %Crude Protein + %Crude Fat + %Crude Fiber).

**Table 2:** The in vivo apparent digestibility (%) of experiment diets (Mean ± SD).

Item	Weaning		Growing		Finishing	
	W1	W2	G1	G2	F1	F2
Dry matter	82.44 ± 2.18	84.12 ± 2.24	84.17 ± 1.13	81.93 ± 1.95	83.92 ± 1.21	79.02 ± 3.43
Crude protein	82.32 ± 2.84	82.12 ± 2.74	74.70 ± 2.42	73.28 ± 2.02	77.69 ± 1.41	74.54 ± 4.74
Crude fat	70.54 ± 2.13	71.42 ± 2.17	83.76 ± 1.14	83.26 ± 1.98	86.15 ± 1.32	85.61 ± 3.19
Crude fiber	16.23 ± 0.91	18.15 ± 1.80	36.32 ± 1.38	38.27 ± 2.12	45.69 ± 2.57	42.55 ± 1.65

**Table 3:** Effects of digestion methods on odorous compound production during in vitro fermentation.

Diet	In Vitro Digestion Method ^1^/Fermentation Time									
	12 h				48 h				*p*-Value	
	S	D	SD	SEM ^2^	S	D	SD	SEM	Time	Method
	p-Cresol (ppm)									
W1	14.16 ^b^	15.65 ^a^	15.43 ^a^	0.27	14.21 ^b^	14.96 ^ab^	15.80 ^a^	0.29	0.704	0.022
W2	17.66 ^b^	23.80 ^a^	22.22 ^a^	0.75	19.01 ^c^	25.34 ^b^	27.47 ^a^	0.51	0.021	0.015
G1	28.71 ^a^	22.53 ^b^	22.87 ^b^	0.39	30.34 ^a^	23.46 ^b^	21.74 ^b^	0.76	0.351	0.021
G2	21.85	22.23	21.86	0.31	14.17 ^c^	15.44 ^b^	17.04 ^a^	0.73	0.012	0.019
F1	22.30	21.68	20.96	0.65	27.31 ^a^	26.47 ^a^	23.13 ^b^	0.85	0.001	0.005
F2	19.67 ^b^	25.14 ^a^	18.27 ^b^	0.63	33.44 ^a^	19.67 ^c^	25.14 ^b^	0.29	0.021	0.018
	Indole (ppm)									
W1	8.00 ^a^	1.19 ^c^	1.97 ^b^	0.09	7.95 ^a^	0.81 ^c^	2.04 ^b^	0.21	0.384	0.023
W2	10.07 ^a^	2.16 ^c^	4.28 ^b^	0.19	11.17 ^a^	2.28 ^c^	5.10 ^b^	0.30	0.039	0.011
G1	3.87 ^a^	0.89 ^c^	1.67 ^b^	0.07	4.17 ^a^	1.34 ^c^	2.12 ^b^	0.11	0.024	0.012
G2	5.25 ^a^	3.05 ^c^	3.74 ^b^	0.15	3.04 ^b^	0.84 ^b^	2.49 ^a^	0.25	0.008	0.011
F1	2.16 ^a^	0.17 ^c^	1.30 ^b^	0.05	1.38 ^a^	0.58 ^b^	0.61 ^b^	0.26	0.011	0.010
F2	8.34 ^a^	2.85 ^c^	4.38 ^b^	0.20	7.58 ^b^	2.85 ^c^	8.34 ^a^	0.12	0.014	0.013
	Skatole (ppm)									
W1	1.40 ^a^	0.71 ^b^	0.78 ^b^	0.03	1.46 ^a^	0.66 ^c^	0.79 ^b^	0.03	0.589	0.017
W2	0.92 ^ab^	0.84 ^b^	0.97 ^a^	0.03	0.98 ^a^	0.77 ^b^	0.90 ^a^	0.03	0.308	0.015
G1	1.93 ^a^	1.26 ^b^	1.30 ^b^	0.04	2.03 ^a^	1.22 ^b^	1.17 ^b^	0.06	0.601	0.023
G2	5.65 ^a^	5.41 ^b^	5.26 ^b^	0.08	6.11 ^a^	2.75 ^c^	4.13 ^b^	0.21	0.013	0.791
F1	0.77 ^a^	0.59 ^b^	0.60 ^b^	0.02	1.02 ^a^	0.65 ^b^	0.65 ^b^	0.05	0.016	0.003
F2	2.59 ^b^	4.28 ^a^	2.48 ^b^	0.12	5.45 ^a^	2.59 ^c^	4.28 ^b^	0.08	0.027	0.011
	NH_3_ (mM)									
W1	175.38 ^a^	104.27 ^b^	118.63 ^b^	7.29	335.30 ^a^	248.35 ^b^	262.84 ^b^	23.53	0.007	0.015
W2	194.53 ^a^	120.00 ^c^	150.77 ^b^	6.71	334.72 ^a^	203.13 ^b^	290.67 ^a^	18.06	0.005	0.004
G1	275.24 ^a^	193.86 ^b^	198.00 ^b^	6.09	308.64 ^a^	288.34 ^a^	216.27 ^b^	20.42	0.009	0.018
G2	354.00 ^a^	245.18 ^b^	272.24 ^b^	14.42	680.00 ^a^	469.43 ^b^	517.30 ^b^	34.69	0.001	0.002
F1	215.86 ^a^	119.31 ^b^	177.93 ^a^	13.00	315.86 ^a^	155.52 ^b^	298.62 ^a^	19.83	0.013	0.017
F2	288.71 ^a^	155.18 ^c^	223.41 ^b^	10.16	455.88 ^a^	223.17 ^c^	323.61 ^b^	18.06	0.031	0.029

^a,b,c^: different letter indicated significant difference between digestion methods at each fermentation time. ^1^ Digestion method: S = Shaking, D = Dialysis, SD = Shaking (gastric phase) + Dialysis (intestinal phase). ^2^ SEM: Stand error of the means.

**Table 4:** Effects of in vitro digestion methods on gas kinetic parameters after 48 h fermentation.

Diet/ Parameters ^2^	In Vitro Digestion Method ^1^			
	S	D	SD	SEM ^3^
W1				
A (mL)	85.76 ^b^	92.86 ^a^	80.64 ^b^	1.03
B (h^−1^)	1.27 ^a^	1.32 ^a^	1.01 ^b^	0.06
C (h)	3.42 ^c^	8.91 ^a^	5.74 ^b^	0.12
T_max_ (h)	0.63 ^b^	2.00 ^a^	0.05 ^c^	0.07
R_max_ (h^−1^)	13.83 ^a^	7.64 ^c^	9.70 ^b^	0.26
W2				
A (mL)	86.19 ^a^	84.96 ^b^	73.24 ^c^	0.77
B (h^−1^)	1.43 ^b^	2.12 ^a^	1.32 ^c^	0.01
C (h)	6.37 ^c^	9.51 ^a^	7.96 ^b^	0.17
T_max_ (h)	1.93 ^b^	5.82 ^a^	1.77 ^b^	0.05
R_max_ (h^−1^)	10.01 ^b^	10.69 ^a^	6.52 ^c^	0.20
G1				
A (mL)	81.29 ^a^	70.06 ^b^	64.84 ^c^	1.43
B (h^−1^)	1.56	1.40	1.52	0.07
C (h)	2.86 ^b^	5.18 ^a^	4.73 ^a^	0.21
T_max_ (h)	1.67 ^ab^	1.09 ^b^	2.25 ^a^	0.21
R_max_ (h^−1^)	9.99 ^c^	16.80 ^a^	10.73 ^b^	0.32
G2				
A (mL)	95.84 ^a^	77.46 ^b^	73.15 ^c^	0.28
B (h^−1^)	1.90 ^a^	1.06 ^c^	1.26 ^b^	0.02
C (h)	3.88 ^b^	7.72 ^a^	3.90 ^b^	0.13
T_max_ (h)	2.10 ^a^	0.28 ^c^	0.71 ^b^	0.06
R_max_ (h^−1^)	23.41 ^a^	7.02 ^c^	10.94 ^b^	0.39
F1				
A (mL)	107.76 ^a^	102.50 ^b^	80.05 ^c^	1.56
B (h^−1^)	1.25 ^b^	0.84 ^c^	1.35 ^a^	0.02
C (h)	4.25 ^b^	10.28 ^a^	3.10 ^c^	0.28
T_max_ (h)	1.04 ^a^	0.08 ^b^	1.14 ^a^	0.06
R_max_ (h^−1^)	15.86 ^a^	7.27 ^b^	15.62 ^a^	0.34
F2				
A (mL)	91.52 ^a^	89.49 ^a^	75.88 ^b^	1.32
B (h^−1^)	2.39 ^a^	1.71 ^b^	1.49 ^c^	0.02
C (h)	5.99 ^b^	7.12 ^a^	5.42 ^b^	0.15
T_max_ (h)	3.26 ^a^	2.95 ^a^	1.76 ^b^	0.14
R_max_ (h^−1^)	16.69 ^a^	10.94 ^b^	10.46 ^b^	0.30

^a,b,c^: different letter indicated significant difference among digestion methods. ^1^ Digestion method: S = Shaking, D = Dialysis, SD = Shaking (gastric phase) + Dialysis (intestinal phase) ^2^ A, asymptotic gas production; B, switching characteristic of the curve; C, the time at half of the asymptote has been reached; T_max_, the time at R_max_ occurs; R_max_, the maximum rate of gas production. ^3^ SEM: Stand error of the means.

**Table 5:** Correlation between in vivo and in vitro digestibility *.

Item ^2^		In Vitro Digestion Method ^1^/Fermentation Time					
		12 h			48 h		
		S	D	SD	S	D	SD
Dry Matter	r	0.9897	0.9805	0.9204	0.9548	0.9874	0.9790
	*p*-value	0.004	0.009	0.042	0.002	0.009	0.003
	CCC	0.8891	0.9734	0.8295	0.8787	0.9760	0.9530
	95% CI	0.9423	0.9864	0.9101	0.9368	0.9877	0.9759
		0.7918	0.9480	0.6886	0.7734	0.9531	0.9091
Crude protein	r	0.6629	0.9271	0.9073	0.9852	0.9759	0.9671
	*p*-value	0.103	0.028	0.004	0.006	0.012	0.019
	CCC	0.7282	0.8919	0.8493	0.9195	0.9524	0.9353
	95% CI	0.8411	0.9439	0.9209	0.9584	0.9756	0.9667
		0.4948	0.7968	0.7223	0.8467	0.9079	0.8759

^1^ Digestion method: S = Shaking, D = Dialysis, SD = Shaking (gastric phase) + Dialysis (intestinal phase). ^2^ r = Pearson’s correlation coefficient; CCC = concordance correlation coefficient (Consistency: >0.95, high; 0.90–0.95 moderate; <0.90, poor). * For a specific digestion method at a fixed fermentation time point (e.g., Method S at 12 h), the total sample number for CCC calculation was 36 (6 diets × 6 replicates).

**Table 6:** Prediction of the odorous compounds concentration and enzyme activity trend from in vitro fermentation dry matter digestibility (IVFDMD) and in vitro fermentation crude protein digestibility (IVFCPD) after different digestion methods.

Digestion Method ^1^	Predict Equation ^2^	R^2^	*p*-Value
IVFDMD (X), Odorous compound/enzyme (Y)			
p-Cresol (ppm)			
S	Y = −0.6180X + 28.3931	0.6502	0.0526
D	Y = −0.7524X + 27.3514	0.8030	0.0156
SD	Y = −1.5017X + 42.3938	0.4039	0.1750
Indole (ppm)			
S	Y = 0.1517X − 3.7155	0.6140	0.0652
D	Y = 0.1510X − 2.7446	0.4527	0.1431
SD	Y = 0.5921X − 12.4926	0.0562	0.6510
Skatole (ppm)			
S	Y = −0.2105X + 7.4484	0.4591	0.1392
D	Y = −0.2786X + 7.5648	0.4792	0.1275
SD	Y = −0.5590X + 13.1917	0.2386	0.3255
NH_3_ (mM)			
S	Y = 2.0825X − 36.0683	0.4494	0.2156
D	Y = 1.4318X − 7.0160	0.8674	0.0214
SD	Y = 4.8709X − 80.4189	0.3646	0.2809
Protease (µg azocasein/mg protein/min)			
S	Y = 0.1032X − 1.6899	0.7771	0.0202
D	Y = 0.1370X − 1.7550	0.8067	0.0150
SD	Y = 0.2134X − 3.2537	0.6668	0.0474
Urease (µmol NH_3_/mg protein/min)			
S	Y = 0.0509X − 0.5430	0.6006	0.0702
D	Y = 0.0566X − 0.3904	0.8531	0.0251
SD	Y = 0.0861X − 0.9191	0.7710	0.0214
IVFCPD (X), Odorous compound/enzyme (Y)			
p-Cresol (ppm)			
S	Y = 0.4946X − 2.6583	0.4963	0.1181
D	Y = 1.4257X − 0.7759	0.3731	0.1977
SD	Y = 0.7457X + 4.3661	0.9595	0.0035
Indole (ppm)			
S	Y = 0.0729X − 1.3713	0.3009	0.2597
D	Y = 0.1165X − 0.2920	0.7369	0.0287
SD	Y = 0.1196X − 0.5820	0.6142	0.0651
Skatole (ppm)			
S	Y = 0.3403X − 8.0163	0.2735	0.2870
D	Y = 0.4904X − 1.9544	0.7790	0.0474
SD	Y = 0.2518X − 0.8230	0.9235	0.0092
NH_3_ (mM)			
S	Y = 0.5163X + 10.0671	0.8233	0.0125
D	Y = 1.1159X + 12.7888	0.8758	0.0061
SD	Y = 1.5326X + 9.0467	0.6670	0.0473
Protease (µg azocasein/mg protein/min)			
S	Y = 0.0767X − 1.0353	0.6878	0.0412
D	Y = 0.1813X − 0.3133	0.7459	0.0266
SD	Y = 0.1737X − 0.7208	0.7363	0.0288
Urease (µmol NH_3_/mg protein/min)			
S	Y = 0.0345X − 0.1246	0.6405	0.0558
D	Y = 0.0754X + 0.2124	0.8381	0.0104
SD	Y = 0.0742X + 0.0587	0.7593	0.0237

^1^ Digestion method: S = Shaking, D = Dialysis, SD = Shaking (gastric phase) + Dialysis (intestinal phase). ^2^ Data from fermentation liquid after 48 h fermentation after different digestion methods.

## Data Availability

The original contributions presented in this study are included in the article. Further inquiries can be directed to the corresponding author.
